# LncRNA AL161431.1 predicts prognosis and drug response in head and neck squamous cell carcinoma

**DOI:** 10.3389/fonc.2023.1134456

**Published:** 2023-06-16

**Authors:** Mingzhu Zhou, Mingyu Mao, Fan Yang, Tao Zhou, Liuqing Zhou, Yuncheng Li

**Affiliations:** ^1^ Department of Otorhinolaryngology, Union Hospital, Tongji Medical College, Huazhong University of Science and Technology, Wuhan, China; ^2^ Department of Neurology, Shanghai Municipal Hospital of Traditional Chinese Medicine, Shanghai University of Traditional Chinese Medicine, Shanghai, China

**Keywords:** AL161431.1, long non-coding RNAs, head and neck squamous cell carcinoma, cell growth, immune cell infiltration, drug sensitivity, prognosis

## Abstract

**Background:**

Long non-coding RNAs (lncRNAs) are increasingly recognized as essential players in various biological processes due to their interactions with DNA, RNA, and protein. Emerging studies have demonstrated lncRNAs as prognostic biomarkers in multiple cancers. However, the prognostic effect of lncRNA AL161431.1 in head and neck squamous cell carcinoma (HNSCC) patients has not been reported.

**Methods:**

In the present study, we conducted a series of analyses to identify and validate the prognostic value of lncRNA AL161431.1 in HNSCC, which included differential lncRNAs screening, survival analysis, Cox regression analysis, time ROCanalysis, nomogram prediction, enrichment analysis, tumor infiltration of immune cells, drug sensitivity analysis, and quantitative real-time polymerase chain reaction (qRT-PCR).

**Results:**

In this study, we performed a comprehensive survival and predictive analysis and demonstrated that AL161431.1 was an independent prognostic factor of HNSCC, for which a high AL161431.1 level indicated poor survival in HNSCC. Functional enrichment analyses found that cell growth and immune-related pathways were significantly enriched in HNSCC, suggesting that AL161431.1 may play a role in tumor development and tumor microenvironment (TME). AL161431.1-related immune cells infiltration analysis demonstrated that AL161431.1 expression is significantly positively associated with M0 macrophages in HNSCC (P<0.001). Using "OncoPredict", we recognized chemotherapy drugs sensitive to the high expression group. Quantitative real-time polymerase chain reaction (qRT-PCR) was performed to identify the expression level of AL161431.1 in HNSCC, and the results further validated our findings.

**Conclusions:**

Our findings suggest that AL161431.1 is a reliable prognostic marker for HNSCC and can potentially be an effective therapeutic target.

## 1 Introduction

Head and neck squamous cell carcinoma (HNSCC), which arises from the mucosal epithelium of the oral cavity, nasopharynx, oropharynx, hypopharynx, and larynx, is the eighth most commonly occurring form of cancer in the world, responsible for approximately 745,000 new cases and 364,340 deaths in 2020 (1). The available clinical treatment modalities for HNSCC include surgery, radiotherapy, chemotherapy, and the latest emerging immunotherapy (2). However, despite a modest improvement in HNSCC survival over the past three decades, the 5-year survival rate still hovers at 60% (3). Therefore, there is an urgent need to find a new molecular biomarker capable of predicting survival, identifying new intervention targets, and predicting response to therapeutic agents.

Long non-coding RNAs (lncRNAs), defined as RNAs longer than 200 nucleotides with minimal coding potential, are becoming a new biomarker for cancer diagnosis and prognosis (4–6). Its abnormal expression is closely associated with the occurrence and progression of HNSCC (7–9). MYOSLID, ZFAS1, KTN1-AS1, MIR31HG, UCA1, NEAT1, EGFR-AS1, and other lncRNAs have been identified as HNSCC-associated prognostic biomarkers (8, 10–15). At the same time, it has been reported that lncRNA AL161431.1 is involved in the occurrence and development of various tumors, including pancreatic cancer (16), endometrial cancer (17), lung squamous cell carcinoma (18), and non-small cell lung cancer (19). However, the prognostic effect of lncRNA AL161431.1 in HNSCC has never been studied. Moreover, the correlation of AL161431.1 with immune infiltration and drug sensitivity in HNSCC has not been determined.

In this study, we conducted a series of analyses, which included differential lncRNAs screening, survival analysis, Cox regression analysis, time ROC analysis, nomogram prediction, enrichment analysis, tumor infiltration of immune cells, drug sensitivity analysis, and quantitative real-time polymerase chain reaction (qRT-PCR). First, we screened the prognosis-related lncRNA AL161431.1 of HNSCC. Then, we confirmed that AL161431.1 was an independent prognostic factor of HNSCC. High expression of AL161431.1 indicated poor survival in HNSCC. Our findings suggest that AL161431.1 has a high potential in the diagnosis, prognosis, and targeted therapy of HNSCC.

## 2 Materials and methods

### 2.1 Data collection and processing

A total of 271 RNA-sequence (17 normal and 254 tumor tissues) data were acquired from the TCGA-HNSCC database. After removing mRNA data, lncRNAs were used for further analysis. In addition, clinical information of 313 HNSCC patients, including age, gender, grade, stage, T-N-M stage, survival time, and survival status, was extracted for subsequent analyses (https://portal. http://gdc.cancer.gov).

### 2.2 Analysis of differentially expressed lncRNAs

The differential lncRNA expression between normal and HNSCC tissues was assessed using the “Limma” package of R software (version 4.0.5) (20). The filtering criteria for lncRNA differential expression were false discovery rate (FDR) < 0.05 and | Log fold change| >1. In addition, the R package “heatmap” was used to display the 100 differentially expressed lncRNAs.

### 2.3 Identification of prognosis-related lncRNAs

Based on the previously obtained differentially expressed lncRNAs, the Kaplan-Meier analysis (KM) and the Cox regression analysis screened the prognosis-related lncRNAs. By merging expression and clinical data, the lncRNAs with P-values < 0.05 were regarded as independent prognostic lncRNAs and were analyzed further. Subsequently, time-dependent receiver operating characteristics (ROC) analysis was conducted to screen out lncRNAs with high accuracy in predicting overall survival (OS) using the “survival”, “survminer”, and “timeROC” R packages.

### 2.4 Differential expression and survival analysis

LncRNA AL161431.1 was selected from the above prognostic lncRNAs for subsequent analyses. The differential analysis used the R package “limma” to investigate the differential expression of AL161431.1 between normal and tumor subgroups. The pan-cancer analysis compared expression values between cancer tissue and adjacent normal tissue by the Wilcoxon rank-sum test (21). More importantly, the expression values of AL161431.1 in HNSCC samples and normal samples were compared. The above results are presented by boxplots. According to the median level, HNSCC tissues were divided into high- and low- AL161431.1 expression groups. Finally, KM analysis compared the OS between the two sets using the “survival” and “survminer” packages in R.

### 2.5 Univariate, multivariate Cox regression and time ROC analysis

The univariate Cox analysis was used to identify the prognostic indicators. At the same time, the multivariate Cox analysis was used to determine whether AL161431.1 is an independent risk factor for OS in HNSCC. First, the ROC curve assessed the diagnostic value of AL161431.1 in HNSCC by the “pROC” R package. Then, the “timeROC” R package was employed to score the prediction accuracy by AL161431.1 using time-dependent ROC curve analysis.

### 2.6 Establishment and evaluation of nomograms

A nomogram encompassing the expression value of AL161431.1 attributes was created using the R packages “rms” and “regplot” to predict HNSCC patient OS (1 year, 2 years, and 3 years). The nomogram’s predictive power was evaluated using calibration curves.

### 2.7 Co-expression and enrichment analyses

The correlation analysis was used to identify the co-expression gene of AL161431.1 in the TCGA-HNSCC cohort to better understand the important biological function that AL161431.1 plays in HNSCC. Then, all co-expression genes that positively and negatively correlated with AL161431.1 were selected for subsequent enrichment analysis. For correlation analysis, the Pearson correlation test was employed. The corresponding coefficient threshold values were set at >0.4. The adjusted P-value was <0.001. The above results were manifested as scatter plots using the “ggplot2” package and R software (version 4.0.5).

Gene Ontology (GO) analyses were carried out to identify the biological effects of AL161431.1 co-expressed genes. In addition, Gene Set enrichment analyses (GSEA) were conducted to identify significant enrichment signaling pathways in the high- and low- AL161431.1 expression groups. The R package “clusterProfiler” was used to evaluate GO analyses and GSEA (22).

### 2.8 Immune cell infiltration

The Cell-type Identification by Estimating Relative Subsets of RNA Transcripts (CIBERSORT) algorithm (23) evaluated immune infiltration in HNSCC tissue in 22 subsets of immune cells, including M0 macrophages, activated dendritic cells, activated mast cells, eosinophils, resting NK cells, CD4 memory resting T cells, neutrophils, M2 macrophages, memory B cells, CD4 naïve T cells, plasma cells, gamma delta T cells, activated NK cells, M1 macrophages, monocytes, CD4 memory activated T cells, naïve B cells, resting dendritic cells, T follicular helper cells (Tfhs), and regulatory T cells (Tregs). Spearman correlation analysis investigated the relationship between AL161431.1 expression and the infiltrated immune cells. Lollipop plots were used to visualize the correlation coefficients of the results (P-value<0.05).

### 2.9 OncoPredict for drug sensitivity analysis

To predict the chemotherapeutic drug sensitivity of the high- and low- AL161431.1 expression groups, we used the “OncoPredict” R package and the Genomics of Drug Sensitivity in Cancer (GDSC; https://www.cancerrxgene.org/) database to predict drug responses in cancer patients (24). First, we analyzed the difference in the activity of drugs between the two groups using the Wilcoxon test. A total of 198 drugs were calculated, and the significance level was set at P <0.001. The “ggplot2” and “ggpubr” functions of R were used to create the box plots.

### 2.10 Validation of the expression level of AL161431.1 in HNSCC by qRT-PCR

A total of 8 HNSCC tissues and 8 adjacent normal mucosal tissues, diagnosed with primary HNSCC after surgical resection in the Wuhan Union Hospital, were collected for subsequent study. There was no history of other tumors, and no patients received radiotherapy, chemotherapy, or other treatments. All patients had signed informed consent before surgery. The collected tissue samples were frozen in liquid nitrogen and stored at -80°C for further RNA extraction. First, total RNA was extracted from samples using TRIzol reagents (Invitrogen, Waltham, MA). Then, reverse transcription and PCR reactions were performed using the PrimeScriptTM RT-PCR kit (Takara, Shiga, Japan). The primer sequence was as follows: AL161431.1:

Forward 5 -TACACTGTTTCTCCAAGCCATCA -3′
Reverse 5 -GAATTGGGAGGATCTAGGACATCTA -3′

### 2.11 Statistical analysis

All analyses were performed using R version 4.0.5, and a P-value<0.0 was considered statistically significant.

## 3 Results

### 3.1 Differential analysis of lncRNA in HNSCC

**Figure 1:**
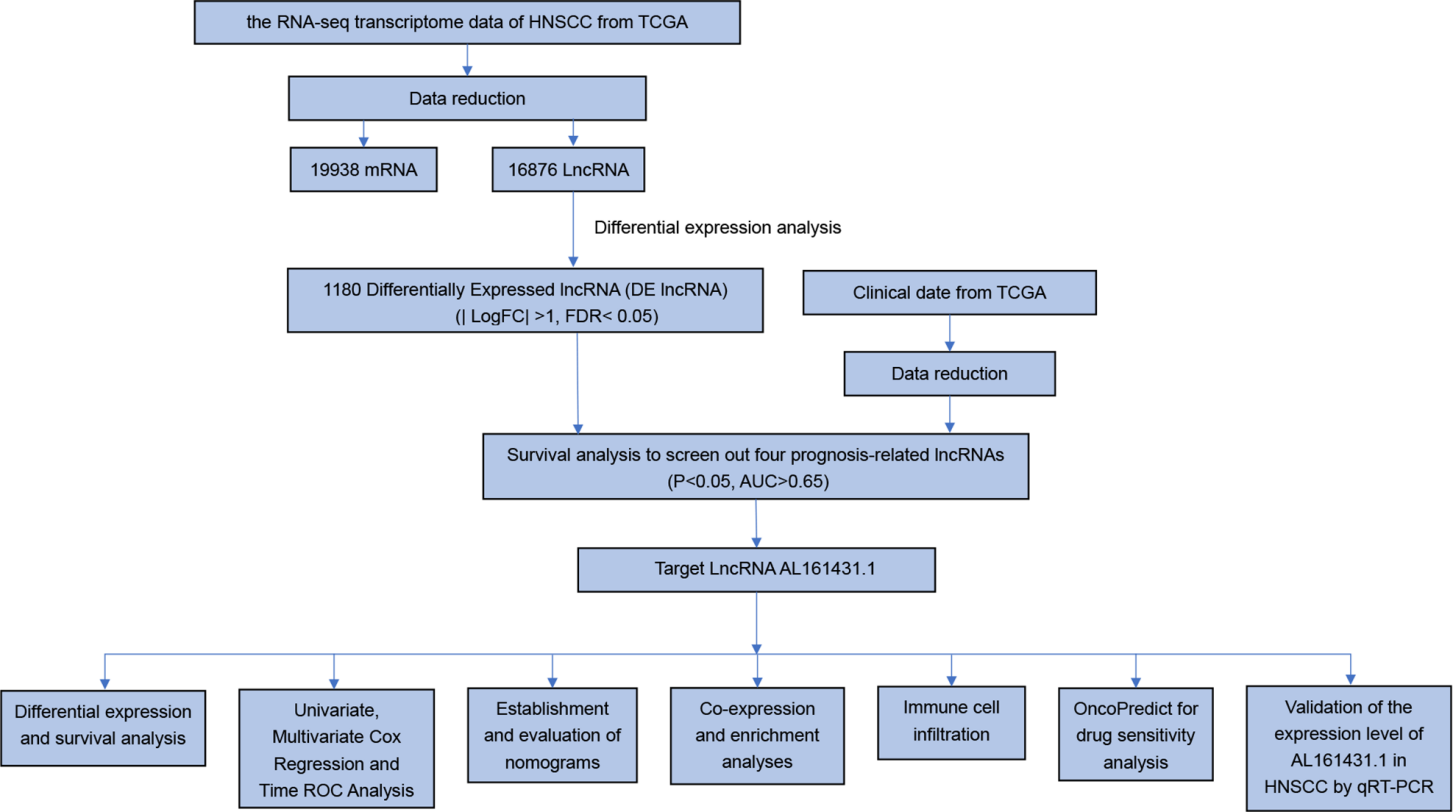
The flow chart of our study.

**Figure 2:**
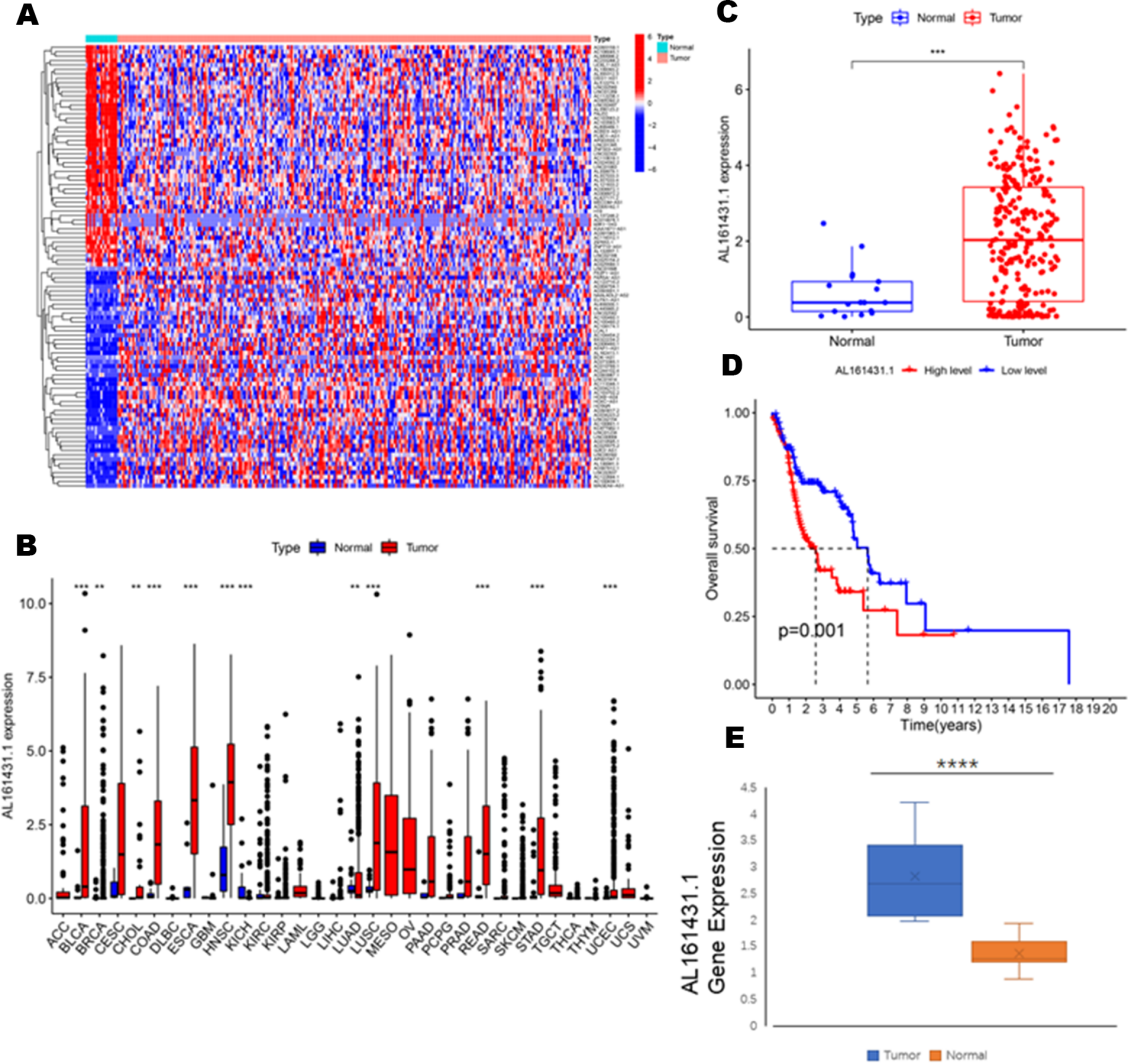
**(A)** Heatmaps of 100 differential lncRNA in normal and tumor samples. **(B)** The scatter diagram indicates the comparative expression of AL161431.1 between pan-cancers and corresponding normal tissues. **(C)** Boxplots present the differential expression of LncRNA AL161431.1 between normal and HNSCC subtypes. **(D)** Kaplan–Meier survival curves show that patients in the AL161431.1 low-level group survived dramatically longer than those in the high-level group. **(E)** Evaluation of the expression of AL161431.1 in HNSCC tissues and adjacent normal tissues (n = 16). The statistical significance is shown as ns, P > 0.05; *P < 0.05; **P < 0.01; ***P < 0.001; ****P < 0.0001.

To identify the expression difference of AL161431.1 in tumors and normal tissues, we analyzed data from 271 patients (17 normal and 254 tumors) extracted from the TCGA-HNSCC database. Following differential expression analysis, 1180 differentially expressed lncRNAs were identified. The study design of this project is shown in **Figure 1**. In addition, the differentially expressed lncRNAs have been presented as a heatmap, including only 100 differentially expressed lncRNAs (**Figure 2A**).

### 3.2 Screening and identification of prognosis-related lncRNAs in HNSCC

To identify lncRNAs associated with the prognosis of HNSCC, we used the Cox regression and KM survival analysis to preliminarily screen out 137 prognostic-related lncRNAs (P <0.05). Then, 81 independent prognostic lncRNAs were identified by independent prognostic analysis (P<0.05). Finally, we used the ROC curve to select four highly accurate lncRNAs in predicting patient survival, namely SNHG26, AL161431.1, LINC00460, and AL358334.2 (AUC>0.65, **Table 1**).

**Table 1:** Four independent prognostic lncRNAs.

lncRNA	SNHG26	AL161431.1	LINC00460	AL358334.2
AUC	0.674476301869521	0.672933769930221	0.67979996009358	0.678265369849573

### 3.3 Expression of AL161431.1 was upregulated in HNSCC and validated by qRT-PCR

To explore the differential expression of AL161431.1 in the normal and tumor samples, we first analyzed its expression in pan-cancer, which included HNSCC. The results demonstrated that compared with normal tissue, the AL161431.1 expression was significantly upregulated in 11 different types of cancer, namely, urothelial bladder carcinoma (BLCA), breast invasive carcinoma (BRCA), cholangiocarcinoma (CHOL), colon adenocarcinoma (COAD), esophageal carcinoma (ESCA), HNSCC, lung adenocarcinoma (LUAD), lung squamous cell carcinoma (LUSC), rectum adenocarcinoma(READ), stomach adenocarcinoma (STAD), and uterine corpus endometrial carcinoma (UCEC) (all P<0.01, **Figure 2B**). In contrast, the AL161431.1 expression was significantly downregulated in kidney Chromophobe (KICH). Further investigation revealed that HNSCC tissues had significantly higher levels of AL161431.1 than normal tissues (P < 0.001, **Figure 2C**). At the same time, KM survival analysis found that high AL161431.1 expression was closely associated with worse OS in HNSCC (P< 0.001, **Figure 2D**). In addition, the expression level of AL161431.1 in HNSCC tissues was verified by qRT-PCR. qRT-PCR analysis showed that AL161431.1 was upregulated in HNSCC compared to normal tissues (**Figure 2E**).

### 3.4 AL161431.1 was an independent prognostic indicator for HNSCC

**Figure 3:**
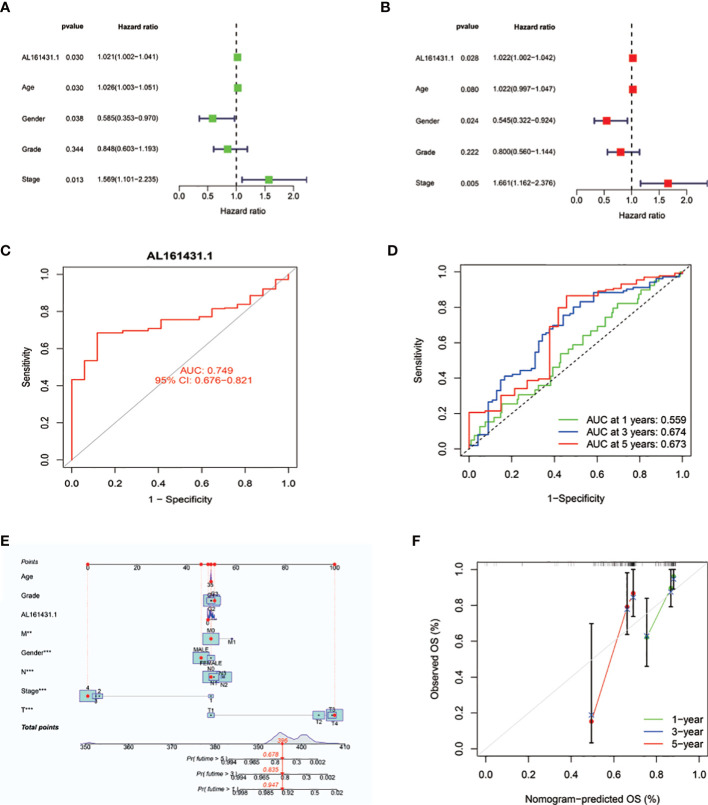
**(A, B)** Univariate and multivariate Cox analyses evaluated the independent prognostic value of AL161431.1 risk signature in HNSCC patients. **(C)** ROC curves show the diagnosis accuracy of AL161431.1. The area under the curve is 0.749. **(D)** ROC curve for AL161431.1 for predicting 1-, 3- and 5-year survival. **(E)** The prediction of 1-, 3-, and 5-year survival for HNSCC patients based on the prognostic nomogram derived from the expression of AL161431.1 and other clinicopathologic features. **(F)** Calibration curve of the nomogram.

We conducted the univariate Cox regression analysis to examine the predictive value of AL161431.1 in HNSCC patients. As illustrated in **Figure 3A**, the result revealed age [HR: 1.026 (1.003-1.051), P = 0.030], gender [HR: 0.585 (0.353-0.970), P = 0.038], stage [HR: 1.569 (1.101-2.235), P =0.013], and AL161431.1 expression [hazard ratio (HR): 1.021 (1.002-1.041), P = 0.030] as the significant predictors of OS in HNSCC patients. After multivariate adjustment, only AL161431.1 [HR: 1.022 (1.002-1.042), P = 0.028], gender [HR: 0.545 (0.322-0.924), P = 0.024], and stage [HR: 1.661(1.162-2.376), P = 0.005] remained significant, as displayed in **Figure 3B**. The results above manifested that AL161431.1 is an independent prognostic factor for HNSCC. As shown in **Figure 3C**, the AUC of AL161431.1 was 0.749, indicating a high diagnostic value of AL161431.1. Furthermore, time-dependent ROC curve analysis revealed that AL161431.1 had a high prognostic value, which the 1 -, 3 -, and 5-year survival rates predicted by the AL161431.1 (AUC) were 0.559, 0.674, and 0.673, respectively (**Figure 3D**).

### 3.5 Construction and assessment of the prognosis nomogram

To precisely estimate the 1-, 3-, and 5-year survival rates, we constructed a nomogram by combining the expression value of AL161431.1 with multiple clinicopathological factors (age, grade, M stage, gender, AJCC stage, N stage, and T stage) (**Figure 3E**). The calibration curve analysis revealed that the predicted and actual 1-, 3- and 5-year survival times were comparable (**Figures 3F**). These findings suggested that the nomogram containing the expression level of AL161431.1 is accurate and reliable.

### 3.6 Co-expression and enrichment analyses reveal that AL161431.1 may involve in the regulation of cell growth

**Figure 4:**
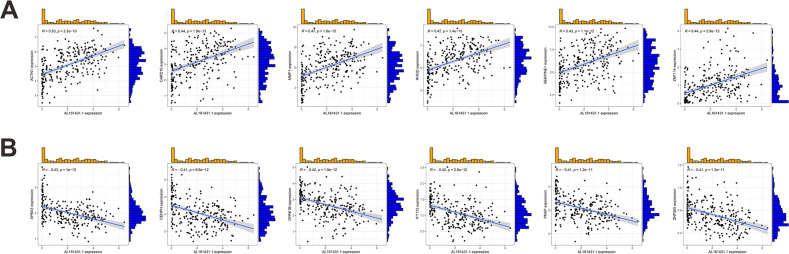
**(A, B)** Scatter plots show the correlation of AL161431.1 expression with the expression of the Co-expression gene. AL161431.1 expression was positively **(A)** or negatively **(B)** correlated with the expression of the Co-expression gene.

**Figure 5:**
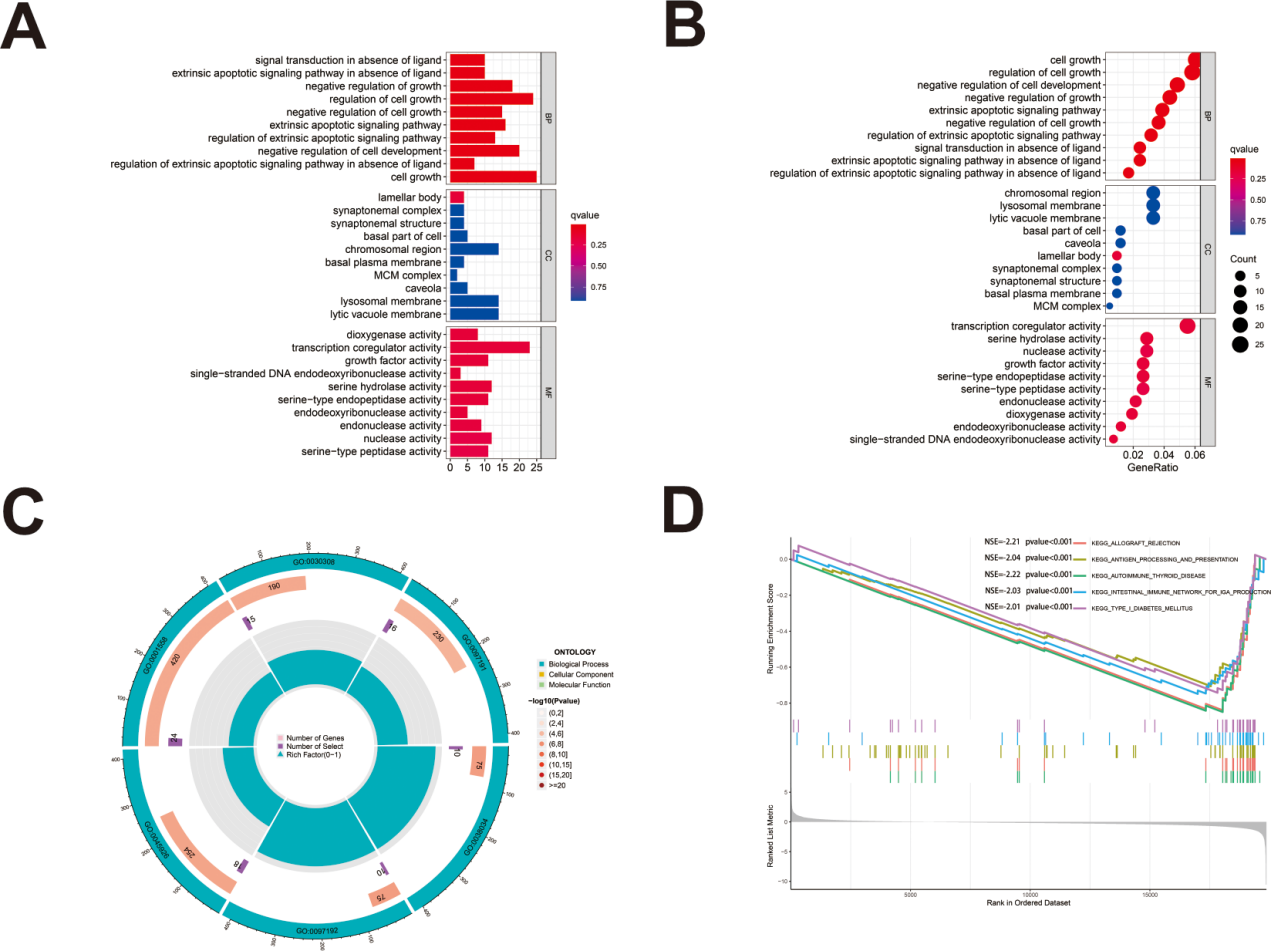
**(A, B)** The bar plot **(A)** and dot plot **(B)** of GO analysis for the top ten enrichments. **(C)** GO analysis was performed to detect biological processes involved in AL161431.1 regulated genes. **(D)** GSEA results show significant enrichment signaling pathways.

We first identified 443 co-expressed genes significantly associated with AL161431.1 to predict the functions and pathways that AL161431.1 may affect (**Figures 4A, B**). Then, we selected these genes to perform GO enrichment analyses, and results revealed AL161431.1 to be mainly associated with cell growth in the biological process (BP) category, the chromosomal region in the cellular component (CC) category, and transcription coregulator activity in molecular function (MF) category (**Figures 5A–C**). In addition, GSEA was conducted to search pathways AL161431.1 might affect. The GSEA results showed that allograft rejection, antigen procession and presentation, autoimmune thyroid disease, the intestinal immune network for IgA production, and type I diabetes mellitus were significantly enriched in the low-AL161431.1 expression group (**Figure 5D**).

### 3.7 The relationship between AL161431.1 expression and the level of immune infiltration in HNSCC

**Figure 6:**
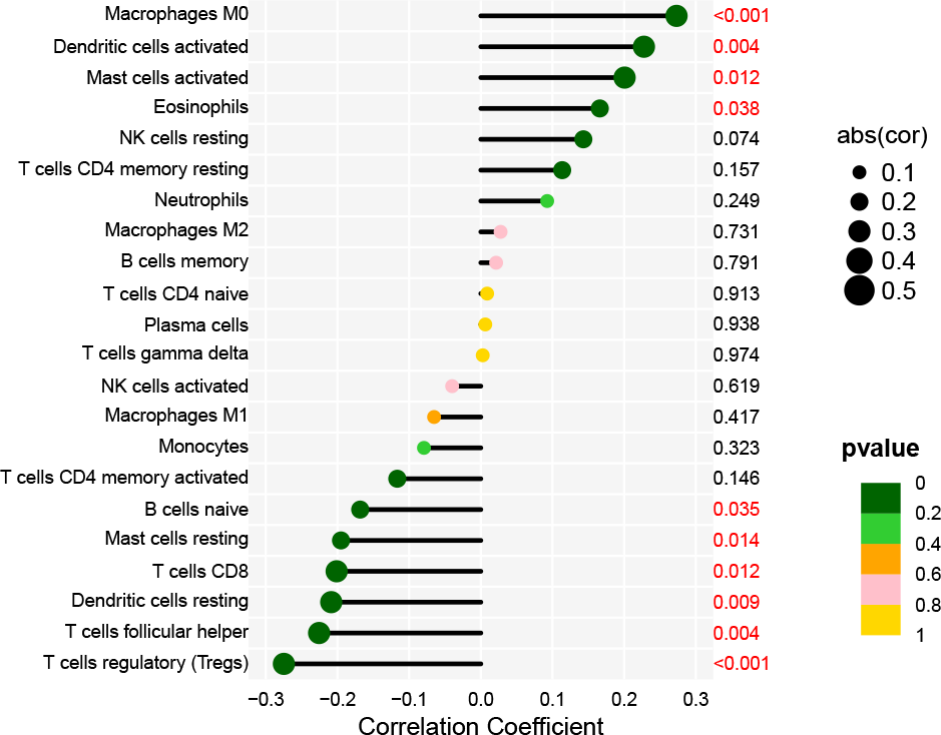
Lollipop charts show the correlation between AL161431.1 and immune cell infiltration in HNSCC samples.

Immune cells that play an essential role in resisting or accelerating tumor growth are tightly linked to the initiation, development, and progression of HNSCC (25). Therefore, we evaluated immune cells significantly associated with AL161431.1 expression and their immune infiltration level in HNSCC. Correlation analysis revealed that the level of infiltration of M0 macrophages, activated dendritic cells, activated mast cells, and eosinophils were significantly and positively correlated with AL161431.1 expression. However, AL161431.1 expression was significantly negatively correlated with the infiltration level of regulatory T cells (Tregs), T follicular helper cells (Tfhs), resting dendritic cells, CD8 T cells, resting mast cells, and naive B cells (all P< 0.05, **Figure 6**).

### 3.8 Drug responses of high- and low- AL161431.1 expression groups in HNSCC

**Figure 7:**
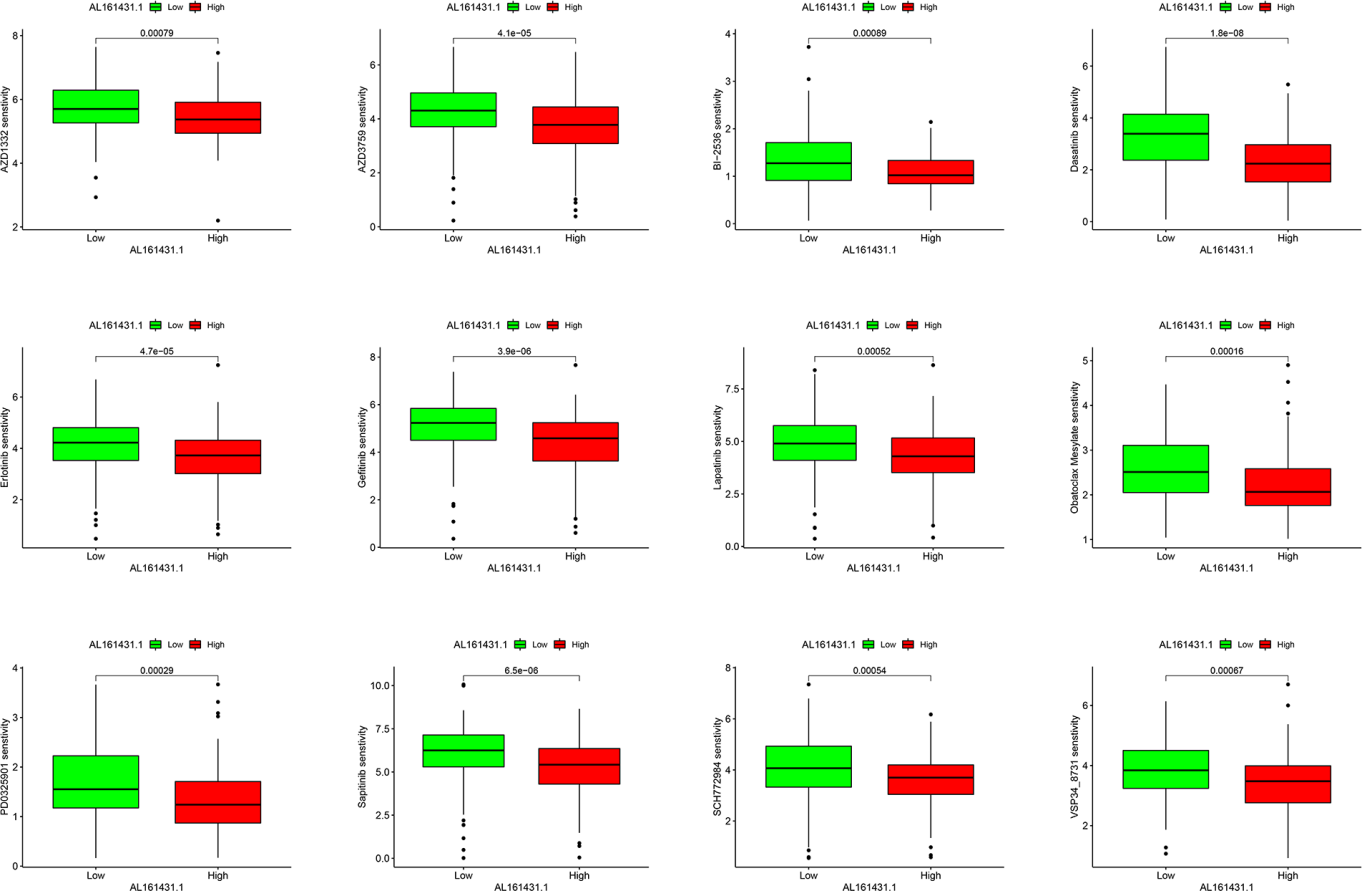
Boxplots present the differences in chemotherapeutic sensitivity between the AL161431.1 low-level and high-level groups.

There were statistically significant response differences between high- and low-AL161431.1 expression groups for 59 of these drugs (P < 0.001). Apart from BI−2536 and PD0325901, several chemotherapeutic drugs, including AZD1332, AZD3759, Dasatinib, Erlotinib, Gefitinib, Lapatinib, Obatoclax Mesylate, Sapitinib, SCH772984, and VSP34_8731, showed a lower score in the high-AL161431.1 expression group (all P< 0.001, **Figure 7**). This suggested that HNSCC patients in the high-AL161431.1 expression group were more likely to respond to these chemotherapeutic drugs.

## 4 Discussion

In the past, the major difficulties in treating HNSCC were late detection, poor response to treatment, and a high recurrence rate. In addition, the molecular heterogeneity of HNSCC has hindered the identification of specific targets and the development of targeted therapy. EGFR inhibitors, as the only approved targeted drugs, have limited efficacy and face the problem of tumor drug resistance (2, 26, 27). In recent years, immunotherapy has brought new hope to patients with HNSCC (28). However, HNSCC remains difficult to treat, with high mortality and poor survival. As a result, determining how to diagnose the disease early and predict the patient’s prognosis is a main priority. With the deepening of research, more and more lncRNAs have been proven to be novel diagnostic and prognostic markers and molecularly targeted therapeutic targets (29–31). This prompted us to search for lncRNAs related to the prognosis of HNSCC to predict patients’ survival and treatment better.

This study comprehensively elucidated the prognostic value of AL161431.1 in patients with HNSCC by bioinformatics methods. We confirmed that the AL161431.1 expression was upregulated in HNSCC neoplastic tissues compared to normal tissues. In addition, we elucidated that AL161431.1 is an independent prognostic indicator for deteriorative OS in HNSCC patients using KM survival analysis combined with Cox regression analysis. Besides, gender and stage were also independent prognostic factors for HNSCC.

We used differentially expressed lncRNA, Kaplan-Meier analysis, and the Cox regression analysis to screen the prognosis-related lncRNAs, which differs from Cao W et al.’s methods, such as the orthogonal partial least squares discrimination analysis (OPLS-DA) and 1.5-fold expression change criterion methods (32). In our study, we first screened out lncRNAs with different expressions in normal and tumor samples. Then, the Kaplan-Meier and Cox regression analyses were performed to screen the prognosis-related lncRNA. However, Cao W et al. first screened out lncRNAs associated with the OS of the patients. Then, the patients were divided into good and poor survival groups based on survival time. Next, the OPLS-DA analysis evaluated the lncRNA expression profile differences between the groups. Finally, the lncRNAs with significant differences between groups were further screened by the 1.5x expression variation criterion.

As a non-parametric estimation method, Kaplan-Meier analysis is currently the most commonly used method for survival analysis. It can analyze the impact of univariate and categorical variables on overall survival and provide a visual representation of survival function (33). Cox regression analysis can handle multiple predictors and confounding variables and provide hazard ratios, which help quantify the effect of predictors on survival (33, 34). Combining these two methods, we established a reliable method for screening the prognosis-related lncRNAs. Many studies have used these two methods to screen prognostic-related lncRNAs and verify the prognostic capability of models. For example, Zhang et al. used differentially expressed lncRNA screening, univariate survival analysis, and multivariate Cox regression analysis to identify a 4-lncRNA signature, which performed well in predicting the prognosis of laryngeal cancer (35). Using univariate and multivariate Cox regression analyses, Wu et al. screened prognosis-related lncRNAs, and an eight-immune-related lncRNA prognostic signature was acquired. Then, Kaplan-Meier analysis and ROC analysis verified the prognostic capability of models (36). Similarly, OPLS-DA and 1.5-fold expression change criterion methods are innovative and effective methods for screening prognostic lncRNAs, which can remove irrelevant variables and effectively separate samples between groups. We used different methods to screen for prognosis-related lncRNAs, but both yielded reliable results.

In previous studies, numerous lncRNAs were aberrantly expressed in various tumors (37, 38). Studies have proved that AL161431.1 is implicated as an oncogene in various tumor types. For example, AL161431.1 was overexpressed in endometrial carcinoma and promoted endometrial carcinoma cell multiplication and migration by activating miR-1252-5p expression (17). Furthermore, LncRNA AL161431.1 could influence the invasion and metastasis of pancreatic cancer cells by promoting the epithelial-mesenchymal transition (EMT) process (16). Studies have also shown that AL161431.1 is an autophagy-related LncRNA, strongly correlated with the prognosis and tumor immune microenvironment of non-small cell lung cancer (19). Shao et al. found that hypoxia-related lncRNA AL161431.1 was highly expressed in LUAD. Inhibition of AL161431.1 can block the proliferation of LUAD cells. The expression level of AL161431.1 was upregulated under hypoxia (39). However, the precise role of AL161431.1 in HNSCC is unknown. In the present study, through GO enrichment analysis, we found that AL161431.1 is involved in a variety of biological functions and processes, such as cell growth, negative regulation of cell growth, and extrinsic apoptotic signaling pathway. In light of these data, AL161431.1 may affect the growth and development of HNSCC, which may be the reason for the poor prognosis of patients with high expression of AL161431.1. However, its specific role and molecular mechanism remain to be studied.

HNSCC is an immunosuppressive disease with high immune infiltration and poor antigen-presenting function (28, 40). The GSEA results in this study showed that several immune-related pathways are enriched in the low-AL161431.1 expression group. Based on these results, we concluded that immunotherapy might be more effective for patients in the low-AL161431.1 expression group than in the high-AL161431.1 expression group.

Immune cells are the cellular basis of immunotherapy. Infiltrating immune cells in tumors is closely related to clinical outcomes and can be used as drug targets for cancer treatment (41). Our correlation analysis of AL161431.1 expression and immune cell infiltration revealed that AL161431.1 expression correlated significantly and positively with M0 macrophages, eosinophils, activated mast cells, and activated dendritic cells. In contrast, regulatory T cells (Tregs), T follicular helper cells (Tfhs), naive B cells, resting mast cells, CD8 T cells, and resting dendritic cells were significantly negatively correlated with it. Studies have shown that lncRNA can activate the expression of immune-cell-related genes, thus leading to tumor immune cell infiltration (42). Meanwhile, immune cells, such as macrophages, dendritic cells, regulatory T cells, and mast cells, are essential to the tumor microenvironment (TME) that can positively or negatively regulate cancer development (43). Macrophages within the TME are called tumor-associated macrophages (TAMs) (44). The latest research has shown that TAMs infiltration often predicts a poor prognosis (45, 46). On the contrary, T cell infiltration, especially CD8 T cells, predicts a favorable prognosis (47). Our study results support these conclusions. Tregs are the most important type of cell in the TME. Typically, Treg cells are dedicated to regulating immune responses to prevent excessive reactivity of the immune system. However, Tregs’ function within the TME is highly complex, as they can promote cancer progression by suppressing the immune response against cancer cells (48). Considerable evidence suggests that Tregs increase during the development of HNSCC, but there is no consensus on the prognostic value of Tregs in HNSCC (49). Several studies strongly support that high levels of Tregs are negatively correlated with the prognosis of HNSCC (50, 51). However, other studies describe a completely different situation, where high Treg numbers are associated with improved overall survival (51, 52). Difficulties in correctly identifying Treg cells may cause these conflicting results. Similarly, this also explains our results that AL161431.1 expression correlated significantly and negatively with Treg cells. Based on these findings, we can better evaluate patients’ prognosis and immunotherapeutic efficacy based on the type and status of infiltrating immune cells.

Hypoxia is a common cause of cancer cell proliferation, metastasis, and recurrence. In addition, hypoxia is closely associated with increased drug resistance in tumors, severely limiting the therapeutic efficacy of HNSCC (53, 54). Increasing data have shown that hypoxic-induced activation of hypoxia-inducible factors (HIFs) can regulate the expression of lncRNA and participate in tumor proliferation, metastasis, and drug resistance (55–57). AL161431.1, a hypoxia-related lncRNA, is upregulated under hypoxia conditions, associated with poor tumor patient survival (39). To improve treatment outcomes, we identified potential chemotherapeutic-sensitive drugs for HNSCC patients with high expression of AL161431.1 by analyzing the differences in sensitivity to chemotherapy drugs between the two sets. We found that patients in the high-AL161431.1 expression group may respond better to chemotherapeutic agents, such as BI−2536, PD0325901, and AZD1332, than those in the low-AL161431.1 expression group. These results may guide the chemotherapy treatment of HNSCC. However, the specific role of AL161431.1 in the difference in chemotherapeutic drug sensitivity in HNSCC warranted further analysis. Finally, we validated the expression of AL161431.1 in clinical specimens by qRT-PCR. Consistent with our previous bioinformatics analysis, the expression of AL161431.1 was upregulated in HNSCC.

To our knowledge, this is the first study on AL161431.1 and HNSCC prognosis, including differential and survival analyses. Our findings suggest that AL161431.1 has a high potential in the diagnosis, prognosis, and targeted therapy of HNSCC. However, our study has several limitations. First, HNSCC has been defined as a highly heterogenous tumor (58). Therefore, the clinical impact of AL161431.1 in other specific subtypes of head and neck cancer remains unclear. Second, we only analyzed the relationship between the AL161431.1 expression and the level of immune infiltration in HNSCC. However, its relationship with the immunosuppressive microenvironment of HNSCC remains to be further studied. Finally, additional experimentation and clinical research are required to further validate the clinical value and potential mechanism of AL161431.1.

## 5 Conclusion

In conclusion, our findings demonstrated that AL161431.1 is highly expressed and associated with a poor prognosis in HNSCC, which might make it a new therapeutic target for HNSCC patients.

## Data availability statement

The datasets presented in this study can be found in online repositories. The names of the repository/repositories and accession number(s) can be found in the article/supplementary material.

## Author contributions

MZ, FY, and LZ conceived and performed bioinformatics analysis. MZ and LZ collected clinical samples. MZ, MM, and FY co-wrote the manuscript. LZ, TZ, and YL undertook a manuscript review. All authors contributed to the article and approved the submitted version.
